# Non-Psychrophilic Methanogens Capable of Growth Following Long-Term Extreme Temperature Changes, with Application to Mars

**DOI:** 10.3390/microorganisms6020034

**Published:** 2018-04-23

**Authors:** Rebecca L. Mickol, Sarah K. Laird, Timothy A. Kral

**Affiliations:** 1 Arkansas Center for Space and Planetary Sciences, University of Arkansas, Fayetteville, AR 72701, USA; rebecca.mickol@gmail.com; 2 American Society for Engineering Education, Washington, DC 20036, USA; 3 Department of Biological Sciences, University of Arkansas, Fayetteville, AR 72701, USA; drsarahlaird@gmail.com

**Keywords:** Mars, methane, methanogens, permafrost, freeze/thaw

## Abstract

Although the martian environment is currently cold and dry, geomorphological features on the surface of the planet indicate relatively recent (<4 My) freeze/thaw episodes. Additionally, the recent detections of near-subsurface ice as well as hydrated salts within recurring slope lineae suggest potentially habitable micro-environments within the martian subsurface. On Earth, microbial communities are often active at sub-freezing temperatures within permafrost, especially within the active layer, which experiences large ranges in temperature. With warming global temperatures, the effect of thawing permafrost communities on the release of greenhouse gases such as carbon dioxide and methane becomes increasingly important. Studies examining the community structure and activity of microbial permafrost communities on Earth can also be related to martian permafrost environments, should life have developed on the planet. Here, two non-psychrophilic methanogens, *Methanobacterium formicicum* and *Methanothermobacter wolfeii*, were tested for their ability to survive long-term (~4 year) exposure to freeze/thaw cycles varying in both temperature and duration, with implications both for climate change on Earth and possible life on Mars.

## 1. Introduction

On Earth, permafrost is defined by temperature and refers to any rocks, soil, or sediments that remain at or below 0 °C for at least two years in a row [1,2]. Permafrost environments typically range between −50 °C and 30 °C, subjecting their microbial communities to large freeze/thaw cycles not encountered elsewhere. On Mars, temperatures vary widely over one sol, often ranging between just above freezing during the day to below −100 °C at night [3], constituting rapid freeze/thaw cycles. Additionally, geomorphological features on the planet such as polygonal patterned ground, thermokarst lakes, and pingo-scars suggest the presence of ice-rich permafrost and point to a warmer, wetter Mars in the relatively recent past [4]. Similarly, Gallagher et al. [5] conclude that high-latitude periglacial landforms are “evidence of the protracted, widespread action of thaw liquids on and within the martian surface” occurring within the last few million years. The authors propose that perchlorate salts, detected by the Phoenix lander, contribute to martian freeze/thaw cycles, resulting in the periglacial geomorphology of the planet [5]. In a separate study, Johnsson et al. [6] also contend that geomorphological features provide evidence for freeze/thaw activity within the last few million years on Mars and suggest that the planet may have a more widespread and cyclic freeze/thaw process than previously thought. 

In the search for life elsewhere in the solar system, and on Mars in particular, methanogens can be considered plausible candidates. Methanogens are microorganisms from the domain Archaea that produce methane (CH_4_) from substrates such as carbon dioxide (CO_2_) and hydrogen (H_2_), among others. Methanogens are often significant members of microbial permafrost communities [1,7,8,9,10,11,12,13] and the release of methane, a potent greenhouse gas, from thawing permafrost is becoming a growing concern amid Earth’s warming temperatures [14,15,16,17,18,19]. Due to their anaerobic nature and ability to use inorganic carbon (e.g., CO_2_) for growth and metabolism, methanogens have also been considered candidates for possible life on Mars. The detection of methane in the martian atmosphere [20,21,22,23,24,25,26,27,28] although controversial [29], also supports the study of methanogens as a possibility for life on Mars. A variety of studies have investigated the growth and survival of specific methanogen species under various martian conditions including low pressure [30,31,32,33,34], salt concentration [35,36,37], and regolith composition [38,39,40,41,42,43,44,45,46], as well as freeze/thaw cycles relevant to Mars [47,48]. 

Recently, Walz et al. [49] monitored the production of carbon dioxide (CO_2_) and methane (CH_4_) from Siberian tundra soils over 150 days in both incubation and freeze/thaw experiments. The authors discovered that methanogenesis was prevalent only within the active layer, with no methane produced within 150 days from permafrost samples. In a 60-day freeze/thaw experiment, Walz et al. [49] found that neither methane production rates nor amounts within soils incubated at 4 °C were significantly different from methane produced by samples incubated at −18 °C for seven days in the middle of the experiment. Thus, a freeze-thaw cycle had no effect on methanogenesis. However, the authors also found that methanogenesis decreased with soil depth and methane production continued to increase over the 150-day incubation period [49]. In a study on methanogenic communities within thawing permafrost in the Tibetan Plateau, Wei et al. [50] discovered that while methanogen abundance does not change in response to freezing or thawing, methanogenesis increases during thaw periods (as evidenced by both methane production and transcriptional activity of the *mcrA* gene [*mcrA* is one of three genes that encode methyl coenzyme M reductase, the catalyst for the terminal step in biogenic methane production, and thus, is present in all methanogens]). Many studies also indicate a shift in the dominant methanogenic orders between frozen and thawed samples, and although methanogenic communities typically vary by environment [15,16,51,52], methanogens within the order Methanosarcinales often comprise the principal order in thawed samples [9,10,50,53,54,55]. Methanogens within the order Methanobacteriales are present in both frozen and thawed permafrost samples at consistent, but low, levels (e.g., 1.3% in frozen samples, 1.8% in thawed samples [50]; see also Coolen and Orsi [15]; Ren et al. [55]; Tveit et al. [18]), providing support for the use of the two methanogens from the order Methanobacteriales in this study. Further support for the use of *Methanobacterium formicicum* is evident in the dominance of a *Methanobacterium* species across three separate ancient permafrost samples tested (19, 27, 33 kyr permafrost, Fox, Alaska; Mackelprang et al. [56]). The existence of methanogens within ancient permafrost samples also has relevance to Mars, should methanogens have arisen earlier on in the planet’s history when conditions were warmer and wetter and may now reside within permafrost. Lastly, an analysis of thawed Alaskan permafrost soils found *Methanosarcina barkeri*, a mesophile, responsible for the majority of methane production (as assessed through *mcrA* transcripts; Coolen and Orsi [15]), which also supports the use of non-psychrophilic methanogens in freeze/thaw studies.

Experiments utilizing freeze/thaw cycles have come under scrutiny for not accurately representing temperature changes seen in nature [57]. However, temperatures can vary widely based on location, season, and whether measurements are taken from the air or soil at varying depths [57]. For example, Zhang et al. [58] analyzed temperature data from 1997 to 1999 within the contiguous United States. The authors discovered that duration of soil freezing ranges between one and eight months, and the number of freeze-thaw cycles that occurs ranges from one to more than eleven, based on location. Additionally, the frozen period of freeze-thaw cycles varied in length from less than twenty days to over 220 days. Thus, over a single season, “a soil freeze/thaw cycle can occur several times, and the length of freeze and thaw within one freeze/thaw cycle may not be symmetric” [58]. As such, previous freeze/thaw experiments have used a variety of cycle lengths, number of cycles, and temperatures (Table 1; see also Henry [57]). Thus, the number of cycles, cycle length, and the temperatures used within the experiments described here, although varied, still reflect important environmental factors possibly encountered by either terrestrial or proposed extraterrestrial microorganisms (such as on Mars).

The experiments conducted here aimed to investigate the effect of long-term (~4 year) freeze/thaw cycles varying in temperature and duration on the methane production of two non-psychrophilic methanogen species within the order Methanobacteriales, *M. formicicum* and *Methanothermobacter wolfeii*. These experiments incorporated the use of varying amounts of liquid medium and sand in order to provide additional stress(es) to the microorganisms and to incorporate environmental factors, in an attempt to mimic a possible subsurface environment.

## 2. Materials and Methods

### 2.1. Microbial Procedures

Methanogens were initially obtained from the Oregon Collection of Methanogens (OCM) (Portland State University, Portland, Oregon). Two methanogen species were cultured in their respective anaerobic growth medium: *M. formicicum* (OCM 55), MS medium supplemented with sodium formate (designated MSF medium) [70] and *M. wolfeii* (OCM 36), MM medium [71]. MSF medium contains the following per liter: 4.0 g NaOH, 1.0 g NH_4_Cl, 1.0 g MgCl_2_·6H_2_O, 0.4 g CaCl_2_·2H_2_O, 0.4 g K_2_HPO_4_·3H_2_O, 1.0 mg resazurin, 5.0 mg Na_2_-EDTA·2H_2_O, 1.5 mg CoCl_2_·6H_2_O, 1.0 mg MnCl_2_©4H_2_O, 1.0 mg FeSO_4_·7H_2_O, 1.0 mg ZnCl_2_, 0.4 mg AlCl_3_·6H_2_O, 0.3 mg Na_2_WO_4_·H_2_O, 0.2 mg CuCl_2_·2H_2_O, 0.2 mg NiSO_4_·6H_2_O, 0.1 mg H_2_SeO_3_, 0.1 mg H_3_BO_3_, 0.1 mg NaMoO_4_·2H_2_O, 2.0 g yeast extract, 2.0 g trypticase peptone, 0.5 g sodium 2-mercaptoethanesulfonate and 0.25 g sodium formate. MM is a minimal medium that contains the same components as MSF medium except yeast extract, trypticase peptone, sodium 2-mercaptoethanesulfonate and sodium formate. All of the media were sparged with 100% CO_2_ gas prior to distribution into test tubes.

For each experiment, growth media were prepared under anaerobic conditions in a 90:10 CO_2_:H_2_ Coy Anaerobic Chamber (Coy Laboratory Products, Inc., Grass Lake Charter Township, MI, USA) following the methods of Kendrick and Kral [72]. In general, 10 mL medium (MSF or MM) were added to each of five anaerobic culture tubes to provide five replicates per set. The tubes were fitted with rubber stoppers and aluminum crimps, sealing the tubes under anaerobic conditions [70]. The media were sterilized via autoclave, after which ~125 μL of 2.5% sodium sulfide (Na_2_S) were added to each tube to remove residual oxygen [70]. Each tube was inoculated with 0.5 mL of the corresponding methanogen culture (MSF medium: *M. formicicum*; MM medium: *M. wolfeii*). The tubes were then pressurized with 2 bar H_2_ and placed at the organisms’ respective incubation temperatures (*M. formicicum*, 37 °C; *M. wolfeii*, 55 °C). Deviations from this standard protocol for each experiment are given in Section 2.2, Section 2.3 , Section 2.4, Section 2.5, below. 

### 2.2. Experiment 1: Growth at 4 °C and 22 °C

Media were prepared as described (see Section 2.1 Microbial Procedures) for each methanogen species. For both species, there were four replicates for each temperature (4 °C and 22 °C). Organisms were inoculated with 0.5 mL culture and test tubes were kept at the desired temperature for the duration of the experiment. Growth was monitored over 140 days by methane production via gas chromatography.

### 2.3. Experiment 2: 5 g Sand, 10 mL Medium

Two types of methanogen growth media (MSF, MM) were prepared as described (see Section 2.1 Microbial Procedures). Two separate sets were prepared (one for each of two organisms) and transfer sets were also prepared as described below. Specific inoculation schemes between original and transfer sets can be found in the supplementary material.

Five grams of sand were added to each of 10 test tubes, with five tubes containing 10 mL MSF medium, and five tubes containing 10 mL MM methanogen growth medium (see Section 2.1 Microbial Procedures). The MSF tubes were inoculated with 0.5 mL of MSF medium containing *M. formicicum* (*n* = 4)*.* The MM test tubes were inoculated with 0.5 mL of MM medium containing *M. wolfeii* (*n* = 4). One test tube for each medium type was not inoculated. After inoculation, each tube was pressurized with 2 bar H_2_ gas. The tubes were next subjected to varying freeze/thaw cycles at temperatures of 55 °C, 37 °C, 22 °C, 4 °C, −15 °C, and −80 °C (Table 2).

After 104 days, a transfer set was prepared following the same method as above. On Day 105, the five transfer tubes with MM medium were each inoculated with 0.5 mL from one tube from the MM Original Set (*n* = 5, Figure S1). The four tubes with MSF medium were each inoculated with 0.5 mL from one tube from the MSF Original Set (*n* = 4, Figure S2). The transfer set was then subjected to various freeze/thaw cycles (Table 2). On Day 179, a second transfer set was prepared following the methods above. In this set, each MM transfer tube was inoculated with 0.5 mL culture from the corresponding MM tube in Transfer Set 1 (Figure S1). Two MSF transfer tubes were inoculated from one tube in MSF Transfer Set and two additional MSF transfer tubes were inoculated from two different tubes in MSF Transfer Set 1 (Figure S2). Tubes within Transfer Set 2 were then subjected to various freeze/thaw cycles (Table 2). On Day 1490, a third transfer set was prepared following the methods above. This set contained only 10 mL medium (either MSF or MM) and did not include any sand. In this set, on Day 1494, each of the tubes (MSF: *n* = 4; MM: *n* = 5) was inoculated with 0.5 mL from the corresponding tube in Transfer Set 2 (Figures S1 and S2). These tubes were incubated at the organisms’ respective growth temperatures (Table 2).

### 2.4. Experiment 3: 10 g Sand, 5 mL Medium

Two types of methanogen growth media (MSF, MM) were prepared, as noted above (see Section 2.1 Microbial Procedures). Two separate sets were prepared (one for each of two organisms) and transfer sets were also prepared as described below. Ten grams of sand were added to each of seven test tubes, with four tubes containing 5 mL MSF medium, and three tubes containing 5 mL MM methanogen growth medium (see Section 2.1 Microbial Procedures, above). The MSF tubes were inoculated with 0.5 mL of MSF medium containing *M. formicicum* (*n* = 4)*.* The MM test tubes were inoculated with 0.5 mL of MM medium containing *M. wolfeii* (*n* = 3). After inoculation, each tube was pressurized with 2 bar H_2_ gas. The tubes were next subjected to varying freeze/thaw cycles at temperatures of 55 °C, 37 °C, 22 °C, 4 °C, −15 °C, and −80 °C (Table 3 and Table 4).

After 90 days, a transfer set was prepared following the same method as above. On Day 91, two transfer tubes with MM medium were each inoculated with 0.5 mL from one tube from the MM Original Set. The remaining transfer tube with MM medium was inoculated with 0.5 mL from a different tube from the MM Original Set (*n* = 3, Figure S3). The four tubes with MSF medium were each inoculated with 0.5 mL from one tube from the MSF Original Set (*n* = 4, Figure S4). This transfer set was then subjected to various freeze/thaw cycles (Table 3 and Table 4). On Day 190, a second transfer set was prepared following the methods above. In this set, three MM tubes were inoculated with 0.5 mL from one MM Transfer Set 1 tube and two MM tubes were inoculated with 0.5 mL from a different MM Transfer Set 1 tube (*n* = 5, Figure S3). Three MSF tubes were inoculated with 0.5 mL from one MSF Transfer Set 1 tube and one MSF tube was inoculated with 0.5 mL from a different MSF Transfer Set 1 tube (*n* = 4, Figure S4). After inoculation, Transfer Set 2 tubes were next subjected to various freeze/thaw cycles (Table 3 and Table 4). On Day 1467, Transfer Set 2 tubes were removed from a −80 °C freezer and thawed at room temperature for seven days (Table 3 and Table 4). A third transfer set was prepared with 10 mL medium (no sand) for both organisms as mentioned above (see Section 2.1 Microbial Procedures). On Day 1474, each tube within Transfer Set 3 was inoculated with 0.5 mL culture from the corresponding tube in Transfer Set 2 (Figures S3 and S4). The tubes were incubated at the organisms’ respective growth temperatures and monitored for methane production. 

### 2.5. Experiment 4: 5 mL Medium

This experiment focused on long-term survival to freeze/thaw cycles in media alone (no sand or regolith). Cultures of *M. formicicum* (*n* = 5) and *M. wolfeii* (*n* = 5) were initially grown in their respective anaerobic growth media (see Section 2.1 Microbial Procedures). Test tubes contained 5 mL media (MSF, *M. formicicum*; MM, *M. wolfeii*) and were inoculated with 0.5 mL culture. Tubes were pressurized with 2 bar H_2_ and incubated at the organisms’ growth temperature (*M. wolfeii*: 55 °C; *M. formicicum*: 37 °C) for 17 days. The cultures were then exposed to varying freeze/thaw cycles for 126 days (Table 5). On Day 126, the cultures were transferred to a −80 °C freezer for 1151 days (3 years, 1 month). On Day 1154, fresh media (10 mL per test tube) were prepared as described above (see Section 2.1 Microbial Procedures). This transfer set was inoculated with 0.5 mL culture from the corresponding tube in the original set on Day 1158 (Figure S5). The tubes were kept at the organisms’ respective growth temperatures for 28 days.

## 3. Results

### 3.1. Experiment 1: Growth at 4 °C and 22 °C

**Figure 1:**
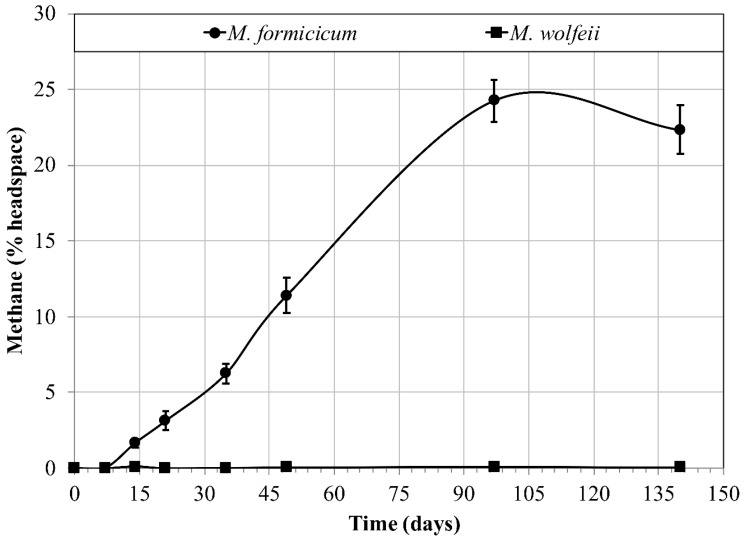
Methane production (% headspace) over time for two species of methanogens (*Methanobacterium formicicum*, *Methanothermobacter wolfeii*) at 22 °C. Error bars indicate ± one standard deviation (*n* = 4).

Methane production did not occur for either methanogen species (*M. formicicum* or *M. wolfeii*) after 140 days’ incubation at 4 °C (data not shown). *M. formicicum* was able to produce methane at 22 °C, however, *M. wolfeii* was not capable of any methane production after 140 days at 22 °C (Figure 1). 

### 3.2. Experiment 2: 5 g Sand, 10 mL Medium

The original sets for both *M. wolfeii* and *M. formicicum* consisted of *n* = 4 replicates (one tube in each set of 5 was not inoculated). Both transfer sets for *M. wolfeii* consisted of *n* = 5 tubes. The transfer sets for *M. formicicum* both consisted of *n* = 4 tubes.

**Figure 2:**
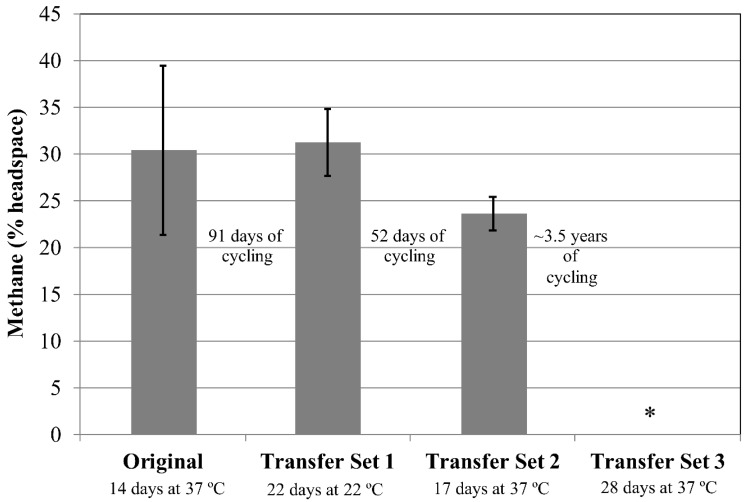
Methane production by *Methanobacterium formicicum* following an initial incubation period for each of three sets. Test tubes for the Original Set and Transfer Sets 1 and 2 contain 5 g sand and 10 mL MSF medium. Transfer Set 3 tubes contain 10 mL MSF medium only. Transfer Set 1 tubes (*n* = 4) were inoculated from one tube in the Original Set (*n* = 4) following 105 days of freeze/thaw cycles. Transfer Set 2 tubes (*n* = 4) were inoculated from three separate test tubes from Transfer Set 1 following 74 days of freeze/thaw cycles. Transfer Set 3 tubes (*n* = 4) were inoculated on Day 1494 from the corresponding tube in Transfer Set 2. Specific freeze/thaw cycles (time, temperature) are given in Table 2. Specific inoculation schemes between sets are given in the supplementary material. The asterisk indicates that no methane was detected. Error bars indicate ± one standard deviation.

Methane production for the four cultures within the original set for *M. formicicum* all displayed ~3% methane production after 5 days (data not shown). After an additional 9 days at 37 °C, methane abundance was much more varied between replicates (~22–42%, Figure 2). After Transfer Set 1 was inoculated and incubated at room temperature for 22 days, methane production within the four replicates ranged between 26% and 34% methane (Figure 2). Additionally, after inoculation and incubation at 37 °C for 17 days, four cultures within Transfer Set 2 displayed methane amounts between 22% and 25% (Figure 2). The lower amount of methane produced by the cultures within Transfer Set 2 may be the result of a lower number of cells in the transfer inoculum and does not necessarily represent cell death. 

**Figure 3:**
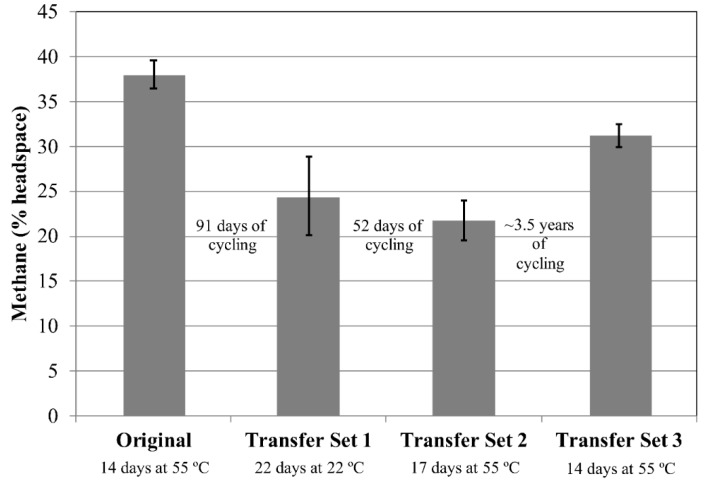
Methane production by *Methanothermobacter wolfeii* following an initial incubation period for each of three sets. Test tubes for the Original Set and for Transfer Sets 1 and 2 contain 5 g sand and 10 mL MM medium. Transfer Set 3 tubes contain 10 mL MM medium only. Transfer Set 1 tubes (*n* = 5) were inoculated from one tube in the Original Set (*n* = 4) following 105 days of freeze/thaw cycles. Transfer Set 2 tubes (*n* = 5) were inoculated from the corresponding tube within Transfer Set 1 following 74 days of freeze/thaw cycles. Transfer Set 3 tubes (*n* = 5 *) were inoculated from the corresponding tube within Transfer Set 2 on Day 1494. Specific freeze/thaw cycles (time, temperature) are given in Table 2. Specific inoculation schemes between sets are given in the supplementary material. Error bars indicate ± one standard deviation. * One replicate did not produce any methane and is not included in the data shown here.

Initial methane production for cultures of *M. wolfeii* reached ~38% methane following 14 days of incubation at 55 °C (Figure 3). After Transfer Set 1 was inoculated, four of the five cultures displayed methane amounts between 2% and 4% after 7 days’ incubation at room temperature (data not shown). After an additional 15 days at room temperature, all five cultures increased in methane abundance, despite the lack of methane production by *M. wolfeii* at 22 °C over 140 days in Experiment 1 (Figure 1), but amounts varied between 17% and 28% (Figure 3). The culture with the lowest methane value (17%) after 22 days corresponds to the culture that showed no methane production (0%) after 7 days’ incubation at room temperature. In the second transfer set, all five cultures displayed methane production after 17 days at 55 °C (19–24%; Figure 3). Similar to the cultures of *M. formicicum*, the decrease in methane amount between the three sets of replicates is not necessarily attributable to cell death and may be the result of fewer cells in the transfer inocula.

This experiment also examined survival following long-term exposure to freezing temperatures. Tubes within Transfer Set 2 were subjected to various freeze/thaw cycles, then kept at −80 °C for ~3 years (Table 2). The tubes were removed from the freezer, thawed at room temperature for 7 days, and then transfers were made to 10 mL fresh media. These new transfer tubes (Transfer Set 3) were stored at the organisms’ respective incubation temperatures and monitored for methane production. For *M. formicicum*, none of the cultures produced methane up to 28 days following inoculation (Figure 2). For *M. wolfeii*, four out of five cultures displayed high methane production (30–33% headspace) after 14 days’ incubation at 55 °C (Figure 3). One culture failed to produce any methane after 14 days’ incubation and is not included in the data shown here. 

### 3.3. Experiment 3: 10 g Sand, 5 mL Medium

**Figure 4:**
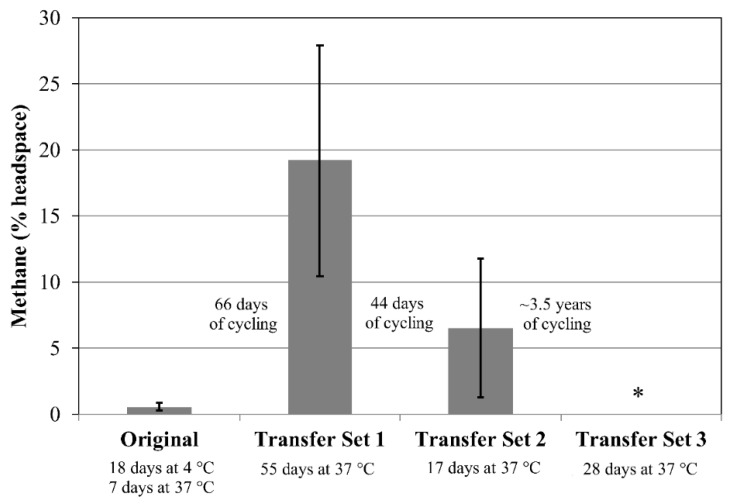
Methane production by *Methanobacterium formicicum* following an initial incubation period for each of three sets. Test tubes for the Original Set and for Transfer Sets 1 and 2 contain 10 g sand and 5 mL MSF medium. Transfer Set 3 tubes contain 10 mL MSF medium only. Transfer Set 1 tubes (*n* = 4) were inoculated from one tube in the Original Set (*n* = 4) following 91 days of freeze/thaw cycles. Transfer Set 2 tubes (*n* = 4) were inoculated from three separate test tubes from Transfer Set 1 following 99 days of freeze/thaw cycles. Transfer Set 3 tubes (*n* = 4) were inoculated from the corresponding tube in Transfer Set 2 after 1474 days of freeze/thaw cycles. Specific freeze/thaw cycles (time, temperature) are given in Table 3. Specific inoculation schemes between sets are given in the supplementary material. The asterisk indicates that no methane was detected. Error bars indicate ± one standard deviation.

There were *n* = 4 tubes each for the Original Set, Transfer Set 1, and Transfer Set 2 for cultures of *M. formicicum*. For *M. formicicum*, methane production was nearly negligible (<1% headspace) for the four tubes within the Original Set after the initial incubation period of 18 days at 4 °C followed by 7 days at 37 °C (Figure 4). In Transfer Set 1, after 55 days’ incubation at 37 °C, three cultures measured 20–26% methane while one measured ~6% methane, resulting in the large error bar for the data shown for Transfer Set 1 (Figure 4). In Transfer Set 2, the four cultures produced varied amounts of methane ranging between 2% and 13% (Figure 4). 

**Figure 5:**
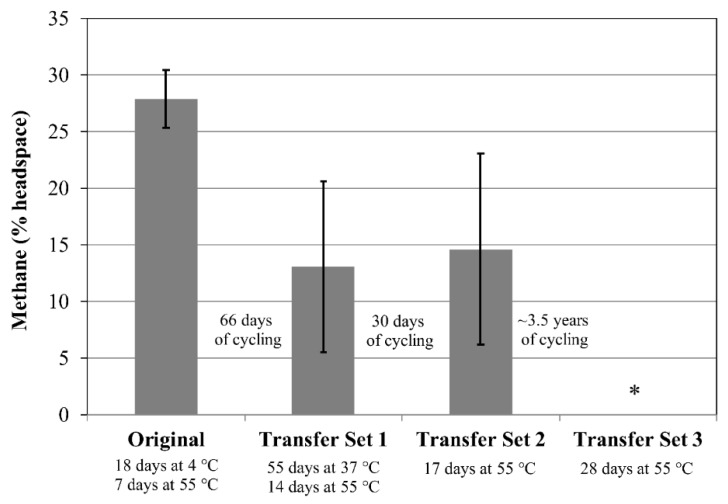
Methane production by *Methanothermobacter wolfeii* following an initial incubation period for each of three sets. Test tubes for the Original Set and for Transfer Sets 1 and 2 contain 10 g sand and 5 mL MM medium. Transfer Set 3 tubes contain 10 mL MM medium only. Transfer Set 1 tubes (*n* = 3) were inoculated from two separate test tubes in the Original Set (*n* = 3) following 91 days of freeze/thaw cycles. Transfer Set 2 tubes (*n* = 5) were inoculated from two separate test tubes from Transfer Set 1 following 99 days of freeze/thaw cycles. Transfer Set 3 tubes (*n* = 5) were inoculated from the corresponding tube in Transfer Set 2 after 1474 days of freeze/thaw cycles. Specific freeze/thaw cycles (time, temperature) are given in Table 4. Specific inoculation schemes between sets are given in the supplementary material. The asterisk indicates that no methane was detected. Error bars indicate ± one standard deviation.

For *M. wolfeii*, three cultures produced an average of ~28% methane following the initial incubation period of 18 days at 4 °C followed by 7 days at 55 °C. In Transfer Set 1 for *M. wolfeii*, the three cultures failed to produce methane following inoculation from the Original Set following 55 days at 37 °C. After an additional 14 days at 55 °C, methane production resumed ranging between 4% and 19% for the three replicates, resulting in the large error bar seen in Figure 5. In Transfer Set 2, of the five cultures for *M. wolfeii*, two produced 22–25% methane after the initial incubation period (17 days at 55 °C), while the other three produced 6–11% methane, resulting in a large error bar for this set as shown in Figure 5. 

It is important to note that the freeze/thaw cycling for Transfer Set 2 for both *M. formicicum* and *M. wolfeii* were not identical (Table 3 and Table 4). The reason for the difference was to allow additional time for *M. wolfeii* cultures to grow, should they be able, after methane production was not seen following the initial incubation period of 55 days at 37 °C for this set.

Replicates within Transfer Set 3 were used to determine if any cells survived freeze/thaw cycling followed by ~3 years at −80 °C. No cultures of either *M. formicicum* or *M. wolfeii* displayed methane production after 28 days’ incubation (Figure 4 and Figure 5). 

### 3.4. Experiment 4: 5 mL Medium 

**Figure 6:**
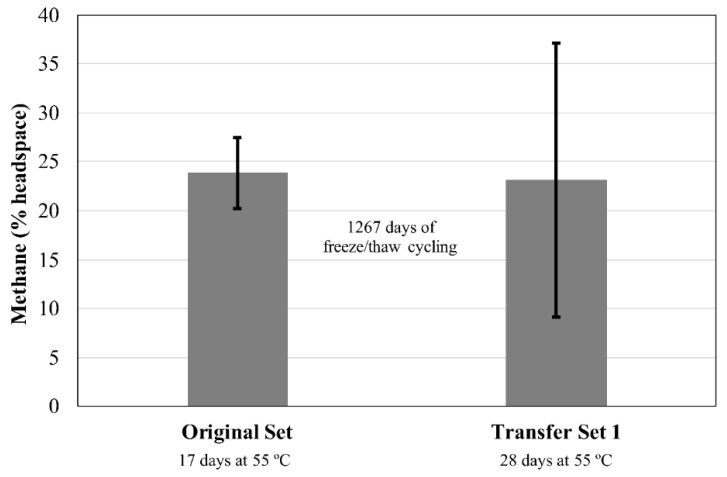
Methane production by *Methanothermobacter wolfeii* following an initial incubation period for each of two sets. Original Set test tubes contained 5 mL MM medium. Transfer Set 1 test tubes contained 10 mL MM medium. Transfer Set 1 tubes (*n* = 5 *) were inoculated from the corresponding replicate in the Original Set (*n* = 5) following 1284 days of freeze/thaw cycles (Table 5). Error bars indicate ± one standard deviation. * Two of five replicates within Transfer Set 1 failed to produce methane after 28 days’ incubation and are not included in the data shown here.

**Figure 7:**
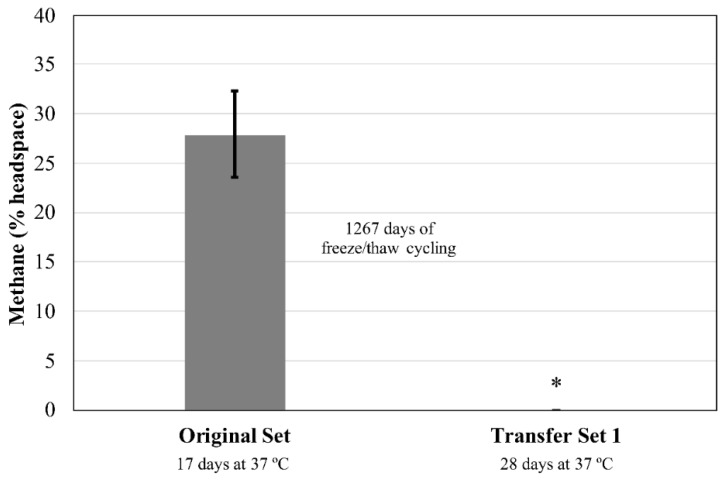
Methane production by *Methanobacterium formicicum* following an initial incubation period for each of two sets. Original Set test tubes contained 5 mL MSF medium. Transfer Set 1 test tubes contained 10 mL MSF medium. Transfer Set 1 tubes (*n* = 5) were inoculated from the corresponding replicate in the Original Set (*n* = 5) following 1284 days of freeze/thaw cycles (Table 5). The asterisk indicates that no methane was detected. Error bars indicate ± one standard deviation.

This experiment aimed to assess survival under long-term freeze/thaw conditions. Tubes were subjected to 126 days of freeze/thaw cycles, then stored at −80 °C. After ~3 years at −80 °C and transfer to fresh media, two out of five cultures of *M. wolfeii* showed appreciable methane production (12.8%, 31.0% headspace) after 14 days’ incubation at 55 °C. The methane abundance within these two cultures increased with another 14 days’ incubation at 55 °C (28.5%, 33.6% methane, respectively), with a third culture producing ~7% methane (Figure 6). The two remaining replicates showed no methane production after 28 days and are not included in the data shown in Figure 6. No methane was produced by cultures of *M. formicicum* after 28 days’ incubation at 37 °C (Figure 7).

## 4. Discussion

### 4.1. Experiment 1: Growth at 4 °C and 22 °C

These data provide a comparison for the subsequent freeze/thaw cycle data. No growth was possible at 4 °C after 140 days for either methanogen, and thus, methane production (cell growth) was not expected during the brief exposures to 4 °C during the subsequent freeze/thaw cycling experiments. Growth was possible at room temperature (22 °C) for *M. formicicum* (Figure 1), but not for *M. wolfeii*, a thermophile, with the highest optimum growth temperature tested here. As such, the subsequent freeze/thaw cycling experiments constitute survival experiments, although the return of active metabolism (methane production, growth) may be possible during brief exposures to 22 °C (for *M. formicicum*) or higher. The optimum temperature for growth for *M. formicicum* is 37 °C, but certain strains are capable of growth down to 20–25 °C [73]. When initially isolated, cultures of *M. wolfeii* did not display growth below 37 °C with optimal growth occurring between 55 °C and 65 °C [74]. Interestingly, in Expt. 2, cultures of *M. wolfeii* displayed methane (>1%) production after only 7 days’ incubation at room temperature, despite the lack of methane production over 140 days at room temperature in Expt. 1 (Figure 1). This phenomenon remains unexplained but does serve to highlight the resiliency of certain species to adapt to changing temperatures. A possible explanation for the growth of cultures of *M. wolfeii* at 22 °C may be due to growth being initiated earlier at a higher temperature, as is required by *Methanococcoides burtonii* (see below; Franzmann et al. [75]). 

Psychrotolerant methanogens exist in pure culture that can actively grow at temperatures down to 1 °C [75,76,77,78,79], but most isolated species have maximum growth temperatures above 23 °C (Table 6; [11,80,81,82,83,84,85,86]). Two exceptions are *Methanogenium frigidum,* which grows maximally at 15 °C with a doubling time of 2.9 days [87] and *Methanolobus psychrophilus*, with an optimum temperature of 18 °C [88]. At low temperatures, the growth rates of both psychrophilic and psychrotolerant species are exceptionally slow such that experiments with these organisms are not conducive to student research timeframes. Additionally, other psychrophilic methanogens, such as *Methanococcoides burtonii*, isolated from Ace Lake in Antarctica, are capable of growth at low temperatures (e.g., 1.7 °C) only after growth is initiated at higher temperature (e.g., 20 °C) [75]. Attempts to culture older stocks of *M. frigidum* at 4 °C using H_2_/CO_2_ with and without acetate were unsuccessful in this lab.

### 4.2. Experiment 2: 5 g Sand, 10 mL Medium, Experiment 3: 10 g Sand, 5 mL Medium, Experiment 4: 5 mL Medium

Experiments 2, 3, and 4 exposed a mesophile, *M. formicicum*, and a thermophile, *M. wolfeii* to a variety of extreme temperature changes over time (Table 2, Table 3, Table 4 and Table 5). As evidenced by Experiment 1, growth was not expected at 4 °C nor 22 °C for *M. wolfeii*. Growth was not expected at 4 °C for *M. formicicum* but was possible at room temperature (22 °C, Figure 1). Thus, the freeze/thaw cycling for Experiments 2 and 3 are considered survival experiments for two non-psychrophilic methanogen species subjected to temperatures between 37 °C or 55 °C and −80 °C (Table 2, Table 3 and Table 4), although resumed metabolism could occur at warmer temperatures. In a study focusing on methanogens in a high Arctic wetland sediment, Blake et al. [51] discovered a strong dependence on temperature for both methane production and methanogen community structure. Additionally, studies indicate that methanogenic permafrost communities may be able to respond rapidly (e.g., within 24 h) to short-term increases in temperature [17,51].

The production of methane within transfer tubes for Experiments 2 and 3 indicates that cells of both *M. formicicum* (Figure 2 and Figure 4) and *M. wolfeii* (Figure 3 and Figure 5) were able to tolerate freeze/thaw cycling and resume active metabolism (methane production) once appropriate temperatures were reached. These studies did not include cell counts and so the specific percentage of survival from the original inoculum is unknown. It also may not be possible to directly compare the amount of methane produced by each set (Original, Transfer 1, Transfer 2, Transfer 3; Figure 2, Figure 3, Figure 4 and Figure 5) based on possible differences in inoculum size (i.e., the exact number of cells contained within 0.5 mL culture that served as inoculum). However, the key point remains that a certain percentage of cells of both *M. formicicum* and *M. wolfeii* were able to tolerate the length and extent of freeze/thaw cycles and could resume metabolism at warmer temperatures in fresh media. 

These experiments also included long-term survival, with tubes exposed to freeze/thaw cycles, then stored at −80 °C for over three years (Table 2, Table 3, Table 4 and Table 5). In Experiment 2, for Transfer Set 3, after transfer to fresh media and 14 days’ incubation at their respective growth temperatures, four out of five replicates of *M. wolfeii* demonstrated high methane production (~30% headspace). This is surprising given the extent of freeze/thaw cycling and the classification of the organism as a thermophile. Replicates of *M. formicicum* subjected to the same conditions failed to produce appreciable methane (>1%) after 28 days’ incubation at 37 °C. In Experiment 3, one replicate of *M. wolfeii* produced 0.70% methane, but all the remaining replicates, as well as the five replicates for *M. formicicum,* failed to produce any methane after incubation at the organisms’ optimal growth temperatures for 28 days. Methane production for cultures of *M. wolfeii* in Experiment 4 was more varied with one replicate measuring 12.8% methane, another replicate measuring 31.0% methane, and three replicates measuring 0% methane after 14 days’ incubation at 55 °C. Cultures of *M. formicicum* in Expt. 4 failed to produce any methane following incubation for 28 days. There are a few possible explanations for this: 1. There were no surviving cells within those cultures (or an insufficient number of cells within cultures before freezing); 2. The rate of freezing was lethal to the microorganisms; or 3. The cells are subject to a significant lag phase and methane production may be delayed. In a study using *M. barkeri*, Gunnigle et al. [91] noticed that growth was much slower at 15 °C compared to 37 °C, and that H_2_/CO_2_ as substrates produced the lowest optical density and methane measurements, compared to methanol as a substrate. However, the strain of *M. barkeri* used was previously adapted for use of methanol as a substrate and that may account for the poor performance of the organism under H_2_/CO_2_ conditions [91]. Additionally, a study that isolated methanogens from permafrost did not see appreciable methane accumulation until 6–12 months of incubation had elapsed, which suggests that longer incubation times could potentially result in resumed metabolism [11]. However, these cultures were enriched from ancient (2.6–5.3 Mya) permafrost and the authors suggest that this long lag phase was likely required to repair damage within cells accumulated over geological time periods, as well as allow time for metabolic adjustment to new environmental conditions [11]. Interestingly, two of the three methanogen species isolated were mesophilic species within the genus *Methanobacterium*. These two species also only grew via hydrogenotrophic methanogenesis (utilizing H_2_ as an energy source and CO_2_ as a carbon source). The two species used in the experiments described here are also solely hydrogenotrophic methanogens. Certain permafrost environments are dominated by hydrogenotrophic methanogenesis [7,18,19,51,52,53,92], although methane produced through the acetoclastic pathway is responsible for up to two-thirds of global methane production [93]. However, Wei et al. [50] interpret a shift in dominant community members from the order Methanomicrobiales to the order Methanosarcinales during permafrost thaw as a shift from hydrogenotrophic methanogenesis to acetoclastic methanogenesis, which could possibly be attributed to renewed availability of acetate. 

To our knowledge, the only other freeze/thaw study using pure cultures of methanogens was conducted by Morozova et al. [48] and focused on survival under simulated martian thermal conditions. This study compared the survival of three psychrophilic methanogens isolated from permafrost against three reference species: *M. barkeri* (a mesophile), *M. frigidium* (isolated from Ace Lake, Antarctica) and *Methanobacterium* spec. MC-20 (isolated from non-permafrost sediments). After exposure to diurnal freeze/thaw cycles between −75 °C and 20 °C for 22 days, the three permafrost strains had the highest survival (60.6–90.4%, cell counts), whereas the survival rate for the reference organisms was exceptionally low (5.8% survival for *M. frigidum*, 1.1% survival for *Methanobacterium* spec. MC-20, 0.3% survival for *M. barkeri*). Additionally, methane production following exposure was significantly decreased for the three reference strains, whereas the permafrost strains had similar methane production before and after exposure [48]. However, methane production following exposure to the freeze/thaw cycles was only monitored for 300 h, which, as evidenced by significant lag times in the experiments described here, as well as experiments using environmental isolates [11], may not be sufficient time for non-psychrophilic organisms, specifically, to resume metabolism following exposure to freeze/thaw cycles.

Sand was utilized in Experiments 2 and 3 to mimic a near-subsurface environment, as may be applicable on Mars. In certain environments, sand also makes up a significant fraction of the permafrost, compared to silt or clay [13]. Additionally, Yang et al. [94] discovered that increased methane emissions correlated with sand content along a tract of thawing permafrost in the Tibetan Plateau. The authors hypothesize that increased sand content fueled higher methane emissions due to three factors: 1. Increased methane diffusion as has been seen in coarser soils; 2. Indirectly affecting methane fluxes through decreased adsorption of H^+^, thereby increasing the pH of the acidic environment; and 3. Indirectly increasing methane emissions by increased water-filled pore space within the soil [94]. Analysis of a permafrost core from the Siberian Arctic discovered lower methane concentrations within sediments containing higher concentrations of sand, citing the greater permeability of coarser-grained soils [14]. Thus, the use of sand within certain experiments has relevance to a possible subsurface environment on Mars, while also reflecting thawing permafrost environments on Earth.

Unlike Earth, Mars is considered to be a planet completely covered in permafrost [95,96]. However, Kreslavsky et al. [97] have stated that an active layer has not been present on Mars in the last 5 My, and thus, that freeze/thaw cycles similar to those on Earth cannot be responsible for the geomorphological features seen on the planet, such as polygonal ground. Additionally, Kreslavsky et al. [97] predict the absence of an active layer in the next 10 My based on martian obliquity calculations. In spite of these predictions, relatively recent findings from the Mars Orbital Camera (MOC) strongly suggest ground ice thaw at low martian latitudes, potentially analogous to the Antarctic polar desert and the low-latitude permafrost zone [98]. Additional analyses of martian surface features using data from MOC, HiRISE, and additional imaging systems also contend that certain landforms on the planet are reminiscent of freeze/thaw geomorphologies seen on Earth [4,5,6]. Baker et al. [98] also suggest that Mars was volcanically active as little as two million years ago, which indicates, at least, local warming and could result in the establishment of habitable environments within thawed permafrost. Ulrich et al. [99] also note periglacial landforms on Mars and suggest that “permafrost is the most promising analog for a potential life habitat on Mars.” Lastly, in reference to habitable environments for methanogens on Mars, Page [100] notes that thawing permafrost could result in the loss of volatiles from the soil and that this could potentially explain the detection of methane plumes over the Cerberus plains on Mars, which feature polygonal ground. 

The reasons for the increased survival of *M. wolfeii* cells under extreme cold temperatures, as compared to the other methanogen tested here, remain unknown. Enhanced survival may be attributable to the presence of DEAD-box RNA helicases, which are believed to function as cold stress proteins within other methanogenic species including the psychrotolerant *M. burtonii* [101], the psychrophile *M. psychrophilus* [102], and the hyperthermophile *Methanococcus jannaschii* [103]. Shimada et al. [104] suggest that DEAD-box RNA helicases allow hyperthermophilic archaea the ability to adapt to lower temperatures. This is evidenced by the presence of these genes within *Thermococcus kodakaraensis*, which typically grows at lower temperatures (optimum growth temperature range for genus *Thermococcus*: 75–95 °C) than the hyperthermophiles within the closely-related genus *Pyrococcus* (optimum growth temperature range: 95–103 °C), which lack any orthologs [104,105]. However, extensive additional research would be required to confirm both the presence and expression of these proteins in *M. wolfeii*. Ding et al. [106] have also demonstrated the up-regulation of a protein disulfide isomerase within *Methanothermobacter thermoautotrophicus* ∆H during growth at temperatures below optimal (50 °C, optimal: 65 °C) and after cold shock at 4 °C. Ultimately, additional experiments are required to assess the true nature of cold-tolerance in *M. wolfeii*.

## 5. Conclusions

The experiments described here subjected a mesophilic and a thermophilic methanogen to long-term exposure (~4 year) to extreme temperature changes varying in duration. The data demonstrate that extreme temperature change (between −80 °C and 37/55 °C) over time is not necessarily lethal to non-psychrophilic methanogens, with resumed metabolism (methane production) possible once warmer temperatures are achieved. These results complement environmental studies, which show that methanogens remain significant community members within both current and ancient permafrost samples, and that methanogenic permafrost communities also feature active, non-psychrophilic species. Should life have arisen on Mars, permafrost may constitute a potentially habitable environment. Further insight into survival and growth in response to extreme temperature changes could be achieved through additional growth proxies such as direct cell counts or transcriptomics analyzing the expression of the *mcrA* gene, as has been performed in certain environmental studies. 

## Figures and Tables

**Table 1:** Examples of previous freeze/thaw studies.

Number of Cycles	Temperatures During Cycles	Length at Temp.	Total Experiment Length	Sample	Reference
22	−75 °C to 20 °C	24 h	22 days	Permafrost and non-permafrost methanogens in pure culture	[48]
18	2 °C −4 °C	9 h 15 h	18 days	Abisko, northern Swedish Lapland, vegetation and soil blocks	[59]
1 or 4	−9 °C and 4 °C	12 h each	1 day or 4 days	Western Alps, the Pennines, alpine soil	[60]
14	−10 °C and 0 °C	12 h each	14 days	Canadian arctic soil	[61]
3	4 °C, 2 °C, 0 °C, −2 °C, −5 °C	24 h each	20 days	Arctic intertidal mud flat sediment	[62]
2	−20 °C and 10 °C	3 weeks each	12 weeks	Arctic intertidal mud flat sediment	[62]
8	−20 °C 10 °C	12 h 18 h	10 days	Arctic intertidal mud flat sediment	[62]
4	−17 °C 4 °C	5 days 7 days	48 weeks	Finnish agricultural soil (loamy sand, peat)	[63]
3	−25 °C 1 °C	15 h 9 h	3 days	Wisconsin soil, seeds of *Elymus canadensis*	[64]
10	−15 °C 17 °C	1 day 6 days	70 days	Canadian grassland soil	[65]
6	5 °C −40 °C	12 min 48 min	6 h	Cloud water isolates	[66]
4	−15 °C, −10 °C, −5 °C, 5 °C, 10 °C	1 week each	8 weeks	Chinese agricultural grassland soil	[67]
3	−5 °C 5 °C	5 days 2 days	21 days	Glas Maol (summit plateau), East Scotland, soil cores	[68]
1	−5 °C	5 days	5 days	Glas Maol soil cores	[68]
1	−5 °C	19 days	19 days	Glas Maol soil cores	[68]
3, 12, 24, 36, or 48 ^a^	10 °C −15 °C	15 h 9 h	12 weeks	Antarctic soil cores	[69]
1	4 °C −18 °C 4 °C	30 days 7 days 30 days	67 days	Lena River Delta, northeast Siberia soil cores	[49]

^a^ Number of cycles corresponds to frequency treatment during a 12-week period: 3 cycles = frequency of one month, 12 cycles = frequency of one week, 24 cycles = 2 cycles per week, 36 cycles = 3 cycles per week, 48 cycles = 4 cycles per week. Cycles were only conducted four out of seven days per week [69].

**Table 2:** Time intervals and temperatures for freeze/thaw cycling for Experiment 2.

			Original Set		Transfer Set 1		Transfer Set 2		Transfer Set 3	
	Cumulative Days of Cycling ^a^	Cumulative Years of Cycling	Time at Temperature (Days)	Temperature ^b^ (°C)	Time at Temperature (Days)	Temperature ^b^ (°C)	Time at Temperature (Days)	Temperature (°C)	Time at Temperature (Days)	Temperature ^b^ (°C)
	**5**	**0.014**	**5**	**37 or 55**						
	**14**	**0.038**	**9**	**37 or 55**						
	**19**	**0.052**	**5**	**4**						
	**26**	**0.071**	**7**	**4**						
	**40**	**0.110**	**14**	**−15**						
	**47**	**0.129**	**7**	**4**						
	**63**	**0.173**	**16**	**−15**						
	**70**	**0.192**	**7**	**4**						
	**77**	**0.211**	**7**	**−15**						
	**96**	**0.263**	**19**	**4**						
	**103**	**0.282**	**7**	**22**	**Inoculated from Original Set on Day 105**					
	**112**	**0.307**			**7**	**22**				
	**127**	**0.348**			**15**	**22**				
	**138**	**0.378**			**11**	**−15**				
	**165**	**0.452**			**27**	**22**				
	**179**	**0.490**			**14**	**4**	**Inoculated from Transfer Set 1 on Day 179**			
	**196**	**0.537**			**17**	**37 or 55**	**17**	**37 or 55**		
	**211**	**0.578**			**15**	**−80**	**15**	**−80**		
	**231**	**0.633**			**20**	**4**	**20**	**4**		
	**252**	**0.690**			**21**	**22**	**21**	**22**		
	**267**	**0.732**			**15**	**−80**	**15**	**−80**		
	**297**	**0.814**					**30**	**4**		
	**334**	**0.915**					**37**	**−15**		
	**1487**	**4.074**					**1153**	**−80**		
	**1494**	**4.093**					**7**	**22**	**Inoculated from Transfer Set 2 on Day 1494**	
	**1508**	**4.132**							**14**	**37 or 55**
	**1522**	**4.170**							**14**	**37 or 55**
**Total**	**1508**	**4.170**	**103**		**162**		**1315**		**28**	

Colors correspond to temperature of cycle: Incubation temperature (red [37 °C, M. formicicum; 55 °C, M. wolfeii]), room temperature (orange, 22 °C), 4 °C (yellow), −15 °C (blue), −80 °C (white). Original set tubes were re-pressurized with 2 bar H_2_ on Day 96 during cycling. ^a^ Cumulative Days of Cycling correspond to the number of days elapsed since the Original Set was first inoculated; ^b^ Instances where temperatures are identical for two adjacent cycles indicate that the cultures were removed from incubation, tested for methane production, and replaced at that temperature for an additional incubation period.

**Table 3:** Time intervals and temperatures for freeze/thaw cycling for Experiment 3 for *Methanobacterium formicicum* cultures.

		Original Set		Transfer Set 1		Transfer Set 2		Transfer Set 3	
Cumulative Days of Cycling ^a^	Cumulative Years of Cycling	Time at Temperature (Days)	Temperature (°C)	Time at Temperature (Days)	Temperature (°C)	Time at Temperature (Days)	Temperature (°C)	Time at Temperature (Days)	Temperature ^b^ (°C)
**18**	**0.049**	**18**	**4**						
**25**	**0.068**	**7**	**37**						
**41**	**0.112**	**16**	**–15**						
**48**	**0.132**	**7**	**4**						
**55**	**0.151**	**7**	**–15**						
**74**	**0.203**	**19**	**4**						
**81**	**0.222**	**7**	**22**						
				**Inoculated from Original Set on Day 91**					
**146**	**0.400**			**55**	**37**				
**160**	**0.438**			**14**	**–15**				
**179**	**0.490**			**19**	**22**				
**196**	**0.537**			**17**	**4**	**Inoculated from Transfer Set 1 on Day 190**			
**207**	**0.567**					**17**	**37**		
**214**	**0.586**			**18**	**–15**				
**228**	**0.625**					**21**	**–80**		
**246**	**0.674**			**32**	**22**				
**284**	**0.778**					**56**	**37**		
**316**	**0.866**					**32**	**–15**		
**1467**	**4.02**					**1151**	**–80**		
**1474**	**4.04**					**7**	**22**	**Inoculated from Transfer Set 2 on Day 1474**	
**1488**	**4.08**							**14**	**37**
**1502**	**4.12**							**14**	**37**
**1502**	**4.08**	**81**		**155**		**1284**		**28**	

Colors correspond to temperature of cycle: Incubation temperature (red, 37 °C), room temperature (orange, 22 °C), 4 °C (yellow), −15 °C (blue), −80 °C (white). Original set tubes were re-pressurized with 2 bar H_2_ on Day 74 during cycling. ^a^ Cumulative Days of Cycling correspond to the number of days elapsed since the Original Set was first inoculated; ^b^ Instances where temperatures are identical for two adjacent cycles indicate that the cultures were removed from incubation, tested for methane production, and replaced at that temperature for an additional incubation period.

**Table 4:** Time intervals and temperatures for freeze/thaw cycling for Experiment 3 for *Methanothermobacter wolfeii* cultures.

		Original Set		Transfer Set 1		Transfer Set 2		Transfer Set 3	
Cumulative Days of Cycling ^a^	Cumulative Years of Cycling	Time at Temperature (Days)	Temperature (°C)	Time at Temperature (Days)	Temperature (°C)	Time at Temperature (Days)	Temperature (°C)	Time at Temperature (Days)	Temperature ^b^ (°C)
**18**	**0.049**	**18**	**4**						
**25**	**0.068**	**7**	**55**						
**41**	**0.112**	**16**	**–15**						
**48**	**0.132**	**7**	**4**						
**55**	**0.151**	**7**	**–15**						
**74**	**0.203**	**19**	**4**						
**81**	**0.222**	**7**	**22**						
				**Inoculated from Original Set on Day 91**					
**146**	**0.400**			**55**	**37**				
**160**	**0.438**			**14**	**55**				
**179**	**0.490**			**19**	**4**				
**196**	**0.537**			**17**	**55**	**Inoculated from Transfer Set 1 on Day 190**			
**207**	**0.567**					**17**	**55**		
**214**	**0.586**			**18**	**−15**				
**228**	**0.625**					**21**	**−80**		
**246**	**0.674**			**32**	**22**				
**284**	**0.778**					**56**	**55**		
**316**	**0.866**					**32**	**−15**		
**1467**	**4.02**					**1151**	**−80**		
**1474**	**4.04**					**7**	**22**	**Inoculated from Transfer Set 2 on Day 1474**	
**1488**	**4.08**							**14**	**55**
**1502**	**4.12**							**14**	**55**
**1502**	**4.12**	**81**		**155**		**1284**		**28**	

Colors correspond to temperature of cycle: Incubation temperature (red, 55 °C), 37 °C (green), room temperature (orange, 22 °C), 4 °C (yellow), −15 °C (blue), −80 °C (white). Original set tubes were re-pressurized with 2 bar H_2_ on Day 74 during cycling. ^a^ Cumulative Days of Cycling correspond to the number of days elapsed since the Original Set was first inoculated; ^b^ Instances where temperatures are identical for two adjacent cycles indicate that the cultures were removed from incubation, tested for methane production, and replaced at that temperature for an additional incubation period.

**Table 5:** Time intervals and temperatures for freeze/thaw cycling for Experiment 4.

			Original Set		Transfer Set 1	
	Cumulative Days of Cycling ^a^	Cumulative Years of Cycling	Time at Temperature (Days)	Temperature (°C)	Time at Temperature (Days)	Temperature ^b^ (°C)
	**17**	**0.47**	**17**	**37 or 55**		
	**38**	**0.104**	**21**	**−80**		
	**94**	**0.258**	**56**	**37 or 55**		
	**126**	**0.345**	**32**	**−15**		
	**1277**	**3.50**	**1151**	**−80**		
	**1284**	**3.52**	**7**	**22**	**Inoculated from Original Set on Day 1284**	
	**1298**	**3.56**	**14**	**37 or 55**	**14**	**37 or 55**
	**1312**	**3.60**			**14**	**37 or 55**
**Total**	**1312**	**3.60**	**1298**		**28**	

Colors correspond to temperature of cycle: Incubation temperature (red, *M. formicicum*: 37 °C, or *M. wolfeii*: 55 °C), room temperature (orange, 22 °C), −15 °C (blue), −80 °C (white). ^a^ Cumulative Days of Cycling correspond to the number of days elapsed since the Original Set was first inoculated; ^b^ Instances where temperatures are identical for two adjacent cycles indicate that the cultures were removed from incubation, tested for methane production, and replaced at that temperature for an additional incubation period.

**Table 6:** Methanogenic archaea isolated from permanently cold environments.

Methanogen	Growth Range (°C)	Optimum Temperature (°C)	Reference
*Methanogenium frigidum* Strain Ace-2^T^	0 ^a^–17	15	[87]
*Methanolobus psychrophilus* Strain R15^T^	0–25	18	[88]
*Methanosarcina baltica* Strain AK-4 *Methanococcoides alaskens* Strain AK-5^T^ Strain AK-9	−2.3 ^b^–28.4 −2.3 ^b^–30.6 −10.7 ^b^–30.1	21 23.6 26	[89]
*Methanococcoides burtonii* Strain DSM6242^T^	−2.54 ^c^–29.5	23.4	[75]
*Methanosarcina lacustris* Strain ZS^T^	1–35	25	[78]
*Methanosarcina baltica* Strain GS1-A^T^	4–27	25	[86]
*Methanogenium marinum* Strain AK-1^T^	5–25	25	[80]
*Methanosarcina soligelidi* Strain SMA-21^T^	0–54	28	[79]
*Methanobacterium movilense* Strain MC-20^T^	0–44	33	[76]
*Methanosarcina subterranea* Strain HC-2^T^	10–40	35	[84]
*Methanobacterium subterraneum* Strain A8p^T^	3.6–45	20–40	[81]
*Methanobacterium aarhusense* Strain H2-LR^T^	5–48	45	[85]
*Methanosarcina lacustris* Strain MM Strain MS *Methanocorpusculum *sp. Strain MSP *Methanomethylovorans hollandica *Strain ZB *Methanosarcina mazei* Strain MT	1–32 1–32 5–35 1–38 5–40	25 25 25 30 35	[77]
*Methanosarcina mazei* Strain JL01 *Methanobacterium veterum* Strain MK4^T^ *Methanobacterium arcticum* Strain M2^T^	10–37 10–45 15–45	24–28 28 37	[11,82,83]

^a^ Growth is possible until medium freezes [87]; ^b^ Based on the Ratkowsky model [89,90]; ^c^ The Ratkowsky model suggests the T_min_ for this species is −2.54 °C, however, cultures incubated at 1.7 °C or 3.2 °C were not capable of growth unless growth was first initiated at 20 °C [75].

## Supplementary Materials

The following are available online at http://www.mdpi.com/2076-2607/6/2/34/s1.
