# Supplementary material for: Non-Psychrophilic Methanogens Capable of Growth Following Long-Term Extreme Temperature Changes, with Application to Mars
Source: Microorganisms. 2018 Apr 23;6(2):34. doi: 10.3390/microorganisms6020034 (PMC6027200; doi:10.3390/microorganisms6020034)
Supplement: Supplementary file 1 [file microorganisms-06-00034-s001.pdf]

Survival of Non-psychrophilic Methanogens Exposed to Extreme Temperature Changes.

Rebecca L. Mickol[1,2], Sarah K. Laird[3], and Timothy A. Kral[1,3]

[1]Arkansas Center for Space and Planetary Sciences, University of Arkansas, Fayetteville, AR;

[2]American Society for Engineering Education, Washington, DC; [3]Dept. of Biological Sciences, University of Arkansas, Fayetteville, AR; [rebecca.mickol@gmail.com]

SUPPLEMENTARY FIGURES

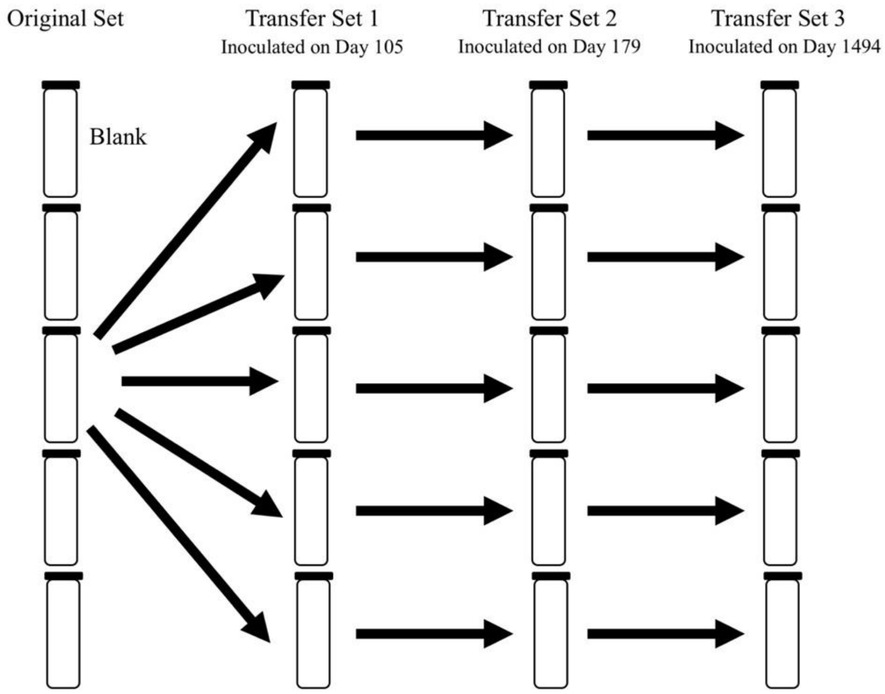

Figure S1. Inoculation scheme for Transfer Sets 1, 2, and 3 for cultures of *Methanothermobacter wolfeii* in Experiment 2 (5 g sand, 10 mL medium).

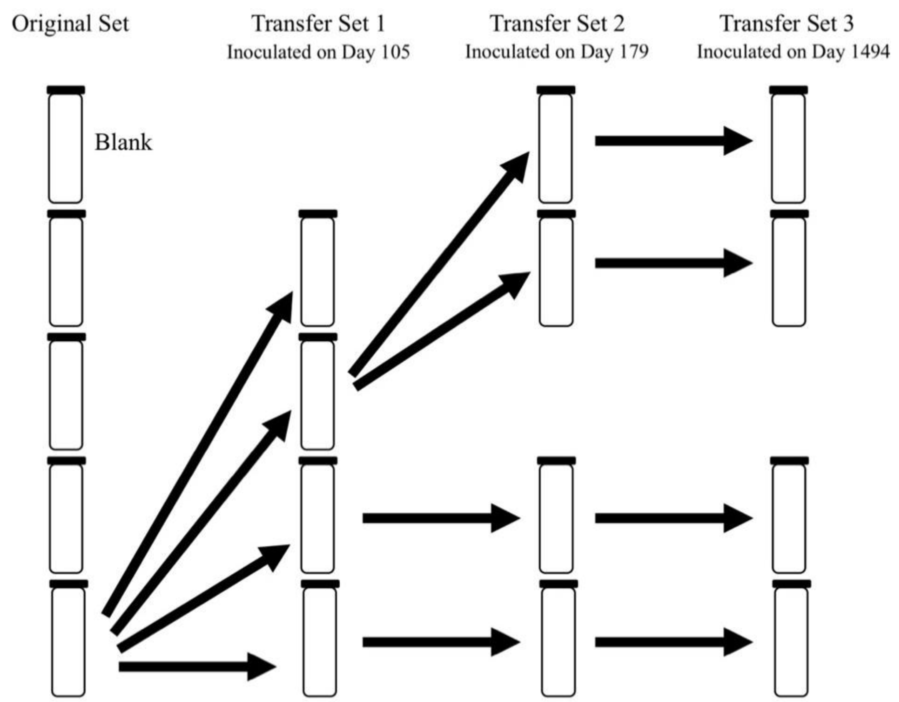

Figure S2. Inoculation scheme for Transfer Sets 1, 2, and 3 for cultures of *Methanobacterium formicicum* in Experiment 2 (5 g sand, 10 mL medium).

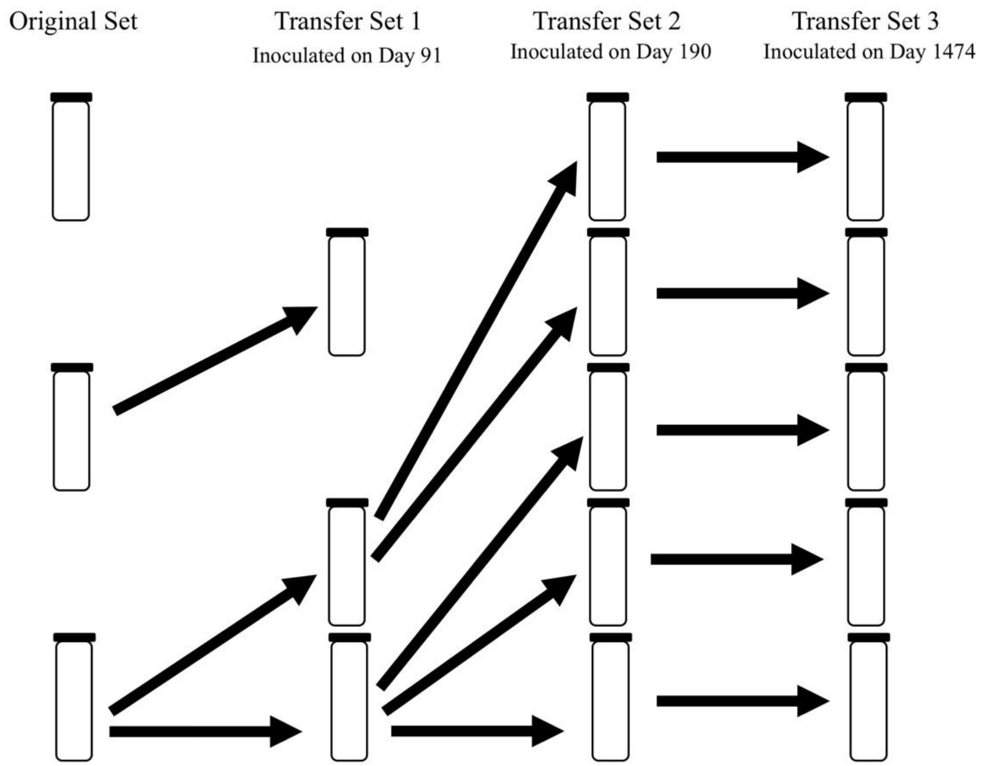

Figure S3. Inoculation scheme for Transfer Sets 1, 2, and 3 for cultures of *Methanothermobacter wolfeii* in Experiment 3 (10 g sand, 5 mL medium).

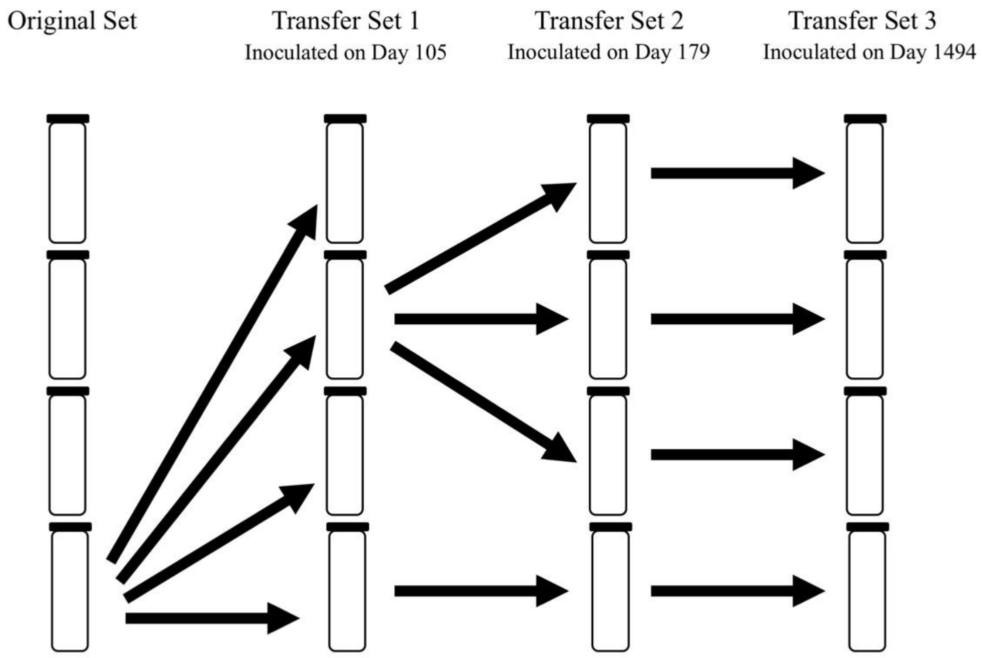

Figure S4. Inoculation scheme for Transfer Sets 1, 2, and 3 for cultures of *Methanobacterium formicicum* in Experiment 3 (10 g sand, 5 mL medium).

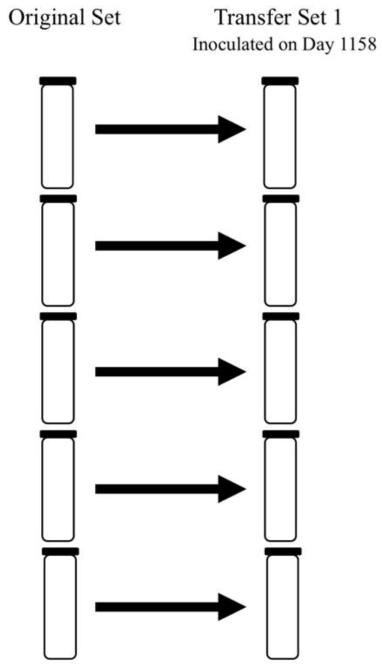

Figure S5. Inoculation scheme for Transfer Sets 1, 2, and 3 for cultures of *Methanobacterium formicicum* and *Methanothermobacter wolfeii* in Experiment 4 (5 mL medium).
